# A functional *IL1RL1* variant regulates corticosteroid-induced sST2 expression in ulcerative colitis

**DOI:** 10.1038/s41598-017-10465-0

**Published:** 2017-08-31

**Authors:** David Díaz-Jiménez, Lucía Núñez, Marjorie De la Fuente, Karen Dubois-Camacho, Hugo Sepúlveda, Martín Montecino, Alejandro Torres-Riquelme, Paulina García-González, Jonás Chnaiderman, Anna Vossenkamper, Thomas T. MacDonald, Daniela Simian, María-Julieta González, John A. Cidlowski, Rodrigo Quera, Marcela A. Hermoso

**Affiliations:** 1 Disciplinary Program of Immunology, Institute of Biomedical Sciences, Faculty of Medicine, Universidad de Chile, Santiago, CL 8380453 Chile; 2 Laboratory of Signal Transduction, National Institute of Environmental Health Sciences, National Institutes of Health, Department of Health and Human Services, Research Triangle Park, North Carolina, USA; 3 Subdirección de Investigación, Dirección Académica, Clínica Las Condes, Santiago, CL 7591018 Chile; 4 Center for Biomedical Research, Faculty of Biological Sciences and Faculty of Medicine, and FONDAP Centre for Genome Regulation, Universidad Andres Bello, Santiago, Chile; 5 Disciplinary Program of Virology, Institute of Biomedical Sciences, Faculty of Medicine, Universidad de Chile, Santiago, CL 8380453 Chile; 6 Centre for Immunobiology, Blizard Institute, Barts and The London School of Medicine and Dentistry, Queen Mary University of London, London, UK; 7 Cell and Molecular Biology Program, Biomedical Sciences Institute, Faculty of Medicine, Universidad de Chile, Santiago, CL 8380453 Chile; 8 Gastroenterology Unit, Clínica Las Condes, Santiago, CL 7591018 Chile

## Abstract

The ST2/IL33 signalling pathway has been associated with ulcerative colitis (UC). ST2, encoded by the *IL1RL1* gene, is expressed as both a membrane-anchored receptor (ST2L) activated by IL33 and as a soluble receptor (sST2) with anti-inflammatory properties. In UC patients, sST2 is further increased by corticosteroid treatment; however, the glucocorticoid-mediated molecular regulation remains unknown. We therefore tested whether genetic variants in the *IL1RL1* distal promoter are involved in UC and affect glucocorticoid-mediated ST2 expression. Serum ST2 levels and genetic variants in the *IL1RL1* distal promoter were examined by ELISA and PCR sequencing in UC patients receiving corticosteroids. Glucocorticoid-mediated ST2 production was evaluated in intestinal mucosa cultures. Molecular regulation of glucocorticoid-mediated ST2 was assessed by RT-qPCR, ChIP assay and luciferase reporter assay. Dexamethasone effect on ST2 transcript expression was analyzed in leukocytes and related to *IL1RL1* variants. Sequencing of a distal *IL1RL1* promoter region demonstrated that SNPs rs6543115(C) and rs6543116(A) are associated with increased sST2 in UC patients on corticosteroids. Dexamethasone up-regulated sST2 transcription through interaction with the glucocorticoid-response element (GRE) carrying rs6543115(C) variant. Our data indicate that *IL1RL1* SNPs rs6543115(C) confer susceptibility to UC and is contained in the GRE, which may modulate glucocorticoid-induced sST2 expression.

## Introduction

Crohn’s disease (CD) and ulcerative colitis (UC) are the most common forms of inflammatory bowel disease (IBD). Both conditions are characterised by chronic gut inflammation, and a relapsing- remitting disease progression. The incidence of IBD worldwide has increased substantially in developed countries the last 50 years^1^.

Evidence to date suggests that a dysregulated mucosal inflammatory response to intestinal antigens in genetically susceptible individuals is involved in IBD^2^. Linkage studies have identified more than 160 single-nucleotide polymorphisms (SNPs) linked to UC and CD, underscoring the genetic complexity of these diseases. The majority of these genetic variants are in genes implicated in mucosal barrier function, innate and adaptive immune responses toward microbiota, endoplasmic reticulum stress, autophagy, metabolic pathways, and other functions^3, 4^.

A locus on chromosome 2q11-2q12, which contains several IL1 receptor superfamily members is associated with UC and CD^5, 6^. *IL1RL1* is located within this gene cluster in a region containing various SNPs with high linkage disequilibrium (LD)^7–9^. Recently, the rs13015714 and rs2058660 SNPs of *IL1RL1* have been shown to contribute to the risk of IBD in an Italian cohort of patients^10^, although no functional significance of these SNPs was demonstrated. Moreover, while SNPs rs6543115(C) and rs6543116(A) have been associated with atopic dermatitis and asthma^7, 8^, an association with UC or CD has not yet been described. In humans, *IL1RL1* expression is regulated by distal and proximal promoters that govern expression of the membrane-anchored receptor (ST2L) activated by IL33, and a soluble isoform (sST2) generated by alternative splicing^11^. sST2 is identical to the ST2L extracellular domain^12^, and is also a decoy receptor for IL33^13^.

It is now well-recognized that the IL33/ST2 signalling pathway is associated with IBD, mainly UC. We previously showed that increased sST2 levels in the gut are related to active disease, and are correlated with serum sST2 levels^14^. Increased sST2 has been proposed to represent a mechanism by which intestinal inflammatory responses are maintained by limiting IL33-driven ST2^+^ T_reg_ cell accumulation and function^15^. In a recent one-year follow-up study of UC patients, we found that sST2 is a biomarker of inflammation and correlates with fecal calprotectin levels^16^. Moreover, UC patients treated with corticosteroids showed increased serum sST2 levels compared with those treated with other agents^14^. Similar to endogenous cortisol, corticosteroid treatment down-regulates inflammatory responses through the glucocorticoid receptor (GR), which suppresses pro-inflammatory cytokines and induces negative regulators of inflammation^17^. Among the many immune cells that contribute to the inflammatory environment of the intestinal mucosa, mast cells are thought to play a role in IBD, based on the increasing evidence that the activity of these cells is not restricted to the first line of defence in infection or as effector cells in allergy^18–22^. Indeed, mast cells are increased in the mucosa of IBD patients and their degranulation results in increased secretion of pro-inflammatory cytokines and other mediators^23^. Although corticosteroids can affect both the number and function of mast cells in IBD^24, 25^, the effects of this therapy on *IL1RL1* expression and on IL33/ST2 signalling have not been described.

Here, we examined whether SNPs rs6543115(C) and rs6543116(A) in the distal *IL1RL1* promoter are linked to UC and whether they are associated with increased sST2 production in UC patients undergoing corticosteroid treatment. We also explored the molecular mechanism leading to glucocorticoid (GC)-induced sST2 production and the impact of SNP rs6543115(C) on this process.

## RESULTS

### UC patients receiving corticosteroid treatment have higher sST2 levels

**Figure 1:**
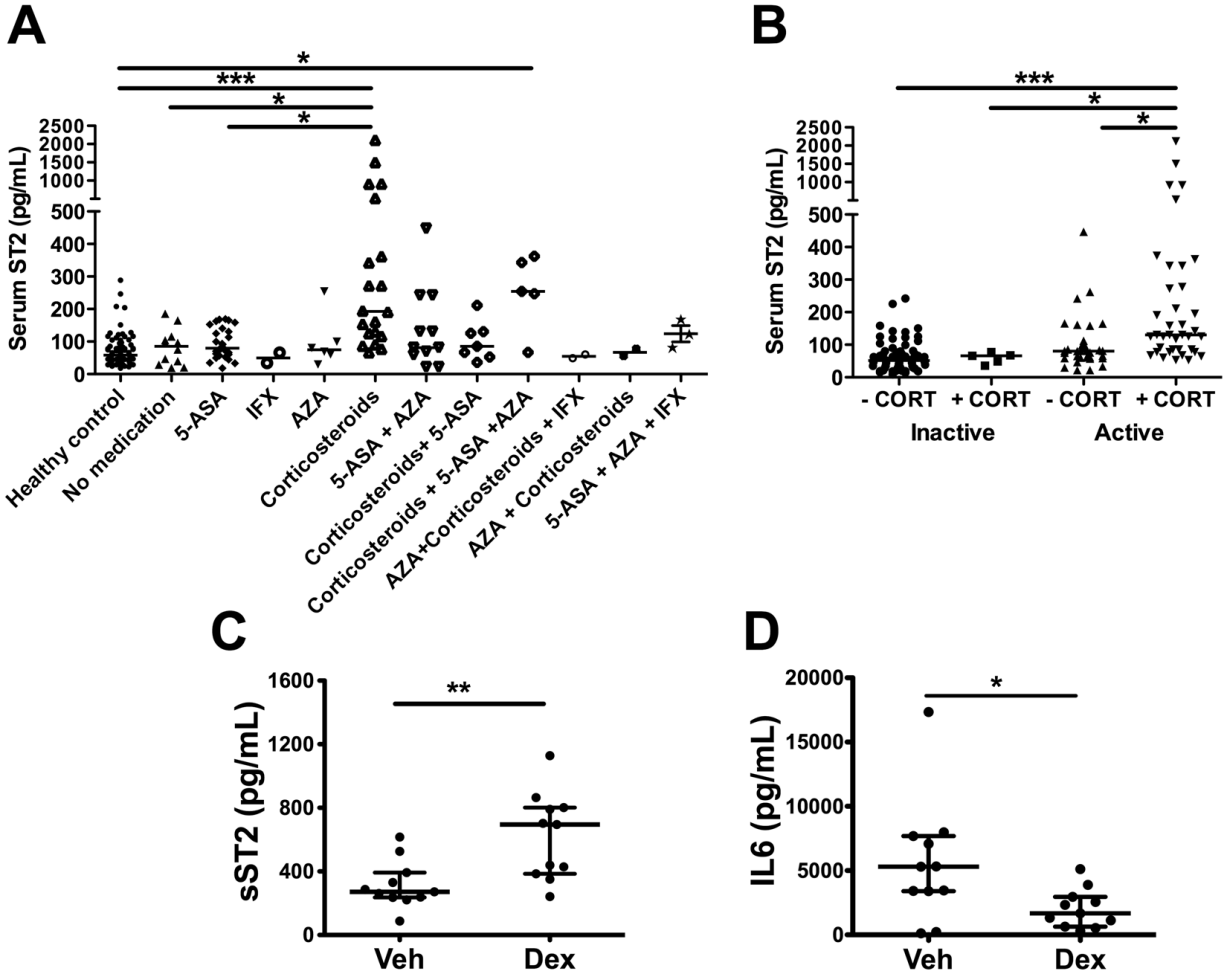
Corticosteroid treatment increases serum levels of ST2 in UC. Serum ST2 levels in UC patients grouped according to therapies (**A**) or according to disease score activity and corticosteroid (CORT) treatment (**B**) were determined by ELISA. 5-ASA (5-aminosalicylic acid), AZA (azathioprine), corticosteroids (hydrocortisone, prednisone and prednisolone), IFX (infliximab). Differences were assessed using the Kruskal-Wallis test and Dunn’s multiple comparison post-test. *p < 0.05; **p < 0.01; ***p < 0.001. sST2 (C), IL6 (**D**) in conditional media of biopsies from healthy controls (HC) or UC patients, stimulated or not with 100 nM Dexametasone (Dex)  for 24 hours. Differences between medians were assessed using the Mann-Whitney U-test. *p < 0.05; **p < 0.01.

Because patients with active UC show increased production of ST2^26^, and are under drug therapy that regulates the inflammatory condition, we tested whether treatment affects sST2 levels. Comparison of serum sST2 levels in a cohort of UC patients in different states of the disease receiving 5-aminosalicylic acid (5-ASA) derivatives, immunosuppressants (azathioprine and mercaptopurine), corticosteroids (hydrocortisone, prednisone and prednisolone), biological treatment (infliximab), combined therapies or no therapy revealed significantly higher sST2 levels in patients receiving corticosteroids either at first presentation or upon disease reactivation, as compared with healthy control (HC) (*p* < 0.001) or those not receiving medication (*p* < 0.05) or those receiving systemic 5-ASA derivatives (*p* < 0.01) (Fig. 1A). On the other hand, CD patients receiving corticosteroid treatment did not show differences in sST2 levels compared to healthy controls or patients under other therapy (Supplementary Figure 1A).

Treatment with corticosteroids is a standard protocol for patients with active UC or CD^27^. In addition, UC patients with active disease have higher sST2 levels than those in remission or with mild disease^14^. Active UC patients treated with corticosteroids (alone or in combination with other therapy) versus active patients receiving a different therapy showed significantly higher serum sST2 levels (Fig. 1B). Moreover, sST2 levels did not vary in active CD patients independently of the received treatment (Supplementary Figure 1B).

### Corticosteroids induce sST2 production in inflamed mucosa

Analysis of the spontaneous production of sST2, IL6, and IL33 and that induced by corticosteroids in culture supernatants of tissue explants (1–3 mm^3^) from inflamed areas of gut of 18 patients with UC and from 10 normal gut of healthy control (Fig. 1C, D and Supplementary Figure 2) revealed no significant difference between control and UC patients in the release of sST2 in unstimulated conditions (HC: median 393.5 pg/ml, range 219.1–570.6 pg/ml; UC: median 266.3 pg/ml, range 232.5–345.6 pg/ml) (Supplementary Figure 2A). Whereas sST2 release after stimulation with Dex was significantly enhanced as compared with unstimulated conditions in UC but not in healthy controls (HC: median 714.3 pg/ml, range 233.4–856.9 pg/ml; UC: median 570.8 pg/ml, range 376.4–817.0 pg/ml; *p* = 0.029) (Fig. 1C and Supplementary Figure 2A). Analysis of the effect of other corticosteroids on sST2 production showed that only prednisone induced the soluble variant of IL33 receptor in comparison with unstimulated conditions in UC patients (*p = *0.0087) but not in controls (*p* > 0.05), although prednisolone did not affect sST2 production as compared with unstimulated conditions either in UC or HC (*p* > 0.05) (Supplementary Figure 2A). IL6 levels, which are elevated in UC mucosa, were also significantly higher in the supernatant of UC biopsies (median 4380 pg/ml, range 2601–7756 pg/ml) compared to those of controls (median 2049 pg/ml, range 1213–2533 pg/ml) (Supplementary Figure 2B). After stimulation with corticosteroids, IL6 was significantly reduced compared to unstimulated conditions in UC but not in healthy controls using Dex (UC: median 1494 pg/ml, range 607.6–2662 pg/ml; *p* = 0.043; HC: median 593.0 pg/ml, range 319.3–1306 pg/ml; *p* > 0.05) (Fig. 1D) and also after stimulation with prednisolone (*p* = 0.041) or prednisone (*p* = 0.047) (Supplementary Figure 2B). No significant changes in IL33 were detected in the same culture supernatants in unstimulated conditions of UC or control mucosa, nor were differences observed in supernatants exposed to any of the corticosteroids used either in UC or healthy mucosa (*p* > 0.05) (Supplementary Figure 2C). These results indicate that corticosteroid-induced sST2 release is independent of tissue damage or necrosis.

Finally, rhIL33 stimulation of tissue mucosa from UC patients or healthy control did not affect sST2 (*p* > 0.05) or IL6 (*p* > 0.05) content in culture supernatants compared with the control vehicle (Supplementary Figure 2D and E).

### Dexamethasone increases sST2 expression in HMC-1 mast cells *in vitro*

During inflammation, various immune-related cells are recruited to the intestinal mucosa. Among these, mast cells, which express both ST2 isoforms^7^, have indeed emerged as important regulators of intestinal epithelial barrier functions^28^. The intestinal mucosa of active UC patients has an increased number of mast cells^29^. and mucosa-infiltrating mast cells staining for both ST2 and tryptase were found (yellow fluorescence in Supplementary Figure 3), suggesting that the inflammatory condition in UC promotes recruitment of tryptase-positive cells expressing ST2.

**Figure 2:**
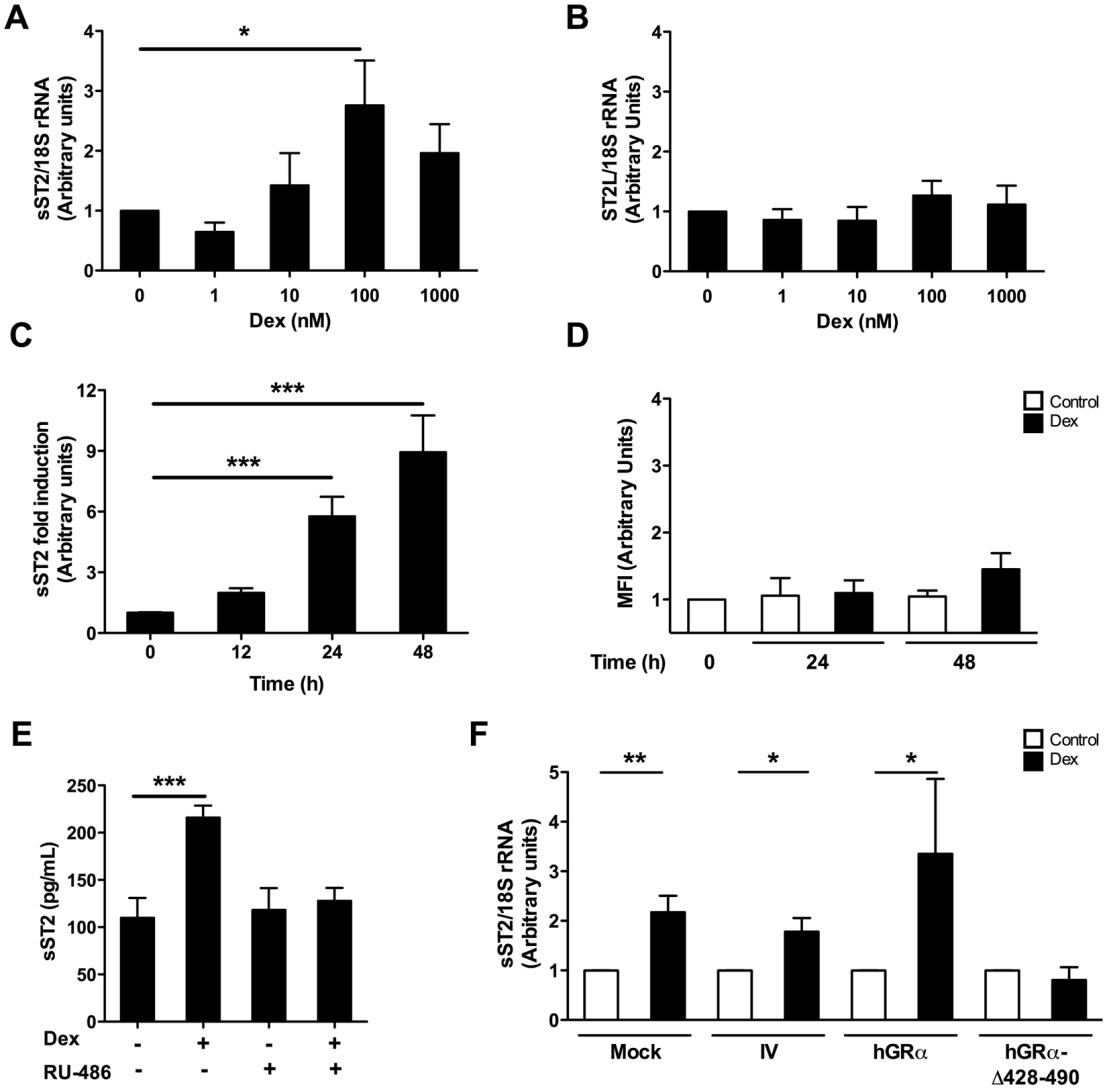
Glucocorticoid-dependent regulation of sST2 expression. Production of (**A**) sST2 and (**B**) aST2L mRNA in HMC-1 cells after treatment for 6 hours with 0–1000 nM Dex, as detected by qPCR (mRNA content normalised to 18 S rRNA). n = 5. *p < 0.05; **p < 0.01. (**C**) sST2 production by HMC-1 cells exposed to 100 nM Dex, as detected in culture supernatant by ELISA. n = 4. ***p < 0.001. (**D**) Surface content of ST2L in HMC-1 cells exposed to 100 nM Dex, as detected by flow cytometry. Mean fluorescence intensity (MFI) was obtained after normalisation to the control condition. n = 3. (**E**) HMC-1 cells stimulated with 100 nM Dex after treatment with or without (+ or −) a receptor antagonist (RU-486) and collected for sST2 ELISA. n = 3. ***p < 0.001. (F) sST2 mRNA expression in HMC-1 cells transduced with lentiviral vectors carrying hGRα, a mutant hGRα (hGRα-Δ428-490), an irrelevant vector (IV), or non-transduced (mock) and treated with 100 nM Dex (mRNA content normalised to 18 S rRNA and compared with the control). Error bars represent the means ± SEM of 4 independent experiments. Kruskal-Wallis test with Dunn’s multiple comparison post-test for each analysis. *p < 0.05; **p < 0.01.

Analysis to determine how glucocorticoids (GCs) affect the function of the GR transcriptional activation pathway in ST2 production by HMC-1 cells stimulated with Dex at a concentration range of 1–1000 nM for 6 hours showed a significant increase in sST2 transcript content after stimulation with 100 nM Dex (*p* = 0.026) (Fig. 2A), but with no affect on ST2L expression (Fig. 2B), suggesting that Dex induces sST2 exclusively. Furthermore, specific expression of the Dex-regulated gene *MKP-1* was also significantly induced by treatments with 1 and 1000 nM Dex (*p* = 0.018 and 0.01, respectively; Supplementary Figure 4A).

The content of sST2 in the culture supernatant of HMC-1 cells increased at 24 hours post-Dex treatment and was sustained up to 48 hours (Fig. 2C) (*p* < 0.001), whereas levels of cell surface ST2L did not differ significantly either at 24 or 48 hours post-Dex treatment (*p* = 0.52; Fig. 2D).

Culture of HMC-1 cells in the presence of RU-486 (mifepristone), an antagonist of GR-mediated effects, abrogated Dex-induced sST2 expression (*p* < 0.001) (Fig. 2E). Likewise, transduction of HMC-1 cells with a human GRα (hGRα) mutant lacking the DNA-binding domain (hGRα Δ428–490) abolished Dex-mediated sST2 transcript expression as compared to control cells transduced with hGRα, an irrelevant vector (IV), or non-transduced (mock) (Fig. 2F). GC-inducible MKP-1 transcript levels were not observed in HMC-1 cells expressing the hGRα Δ428–490 receptor (Supplementary Figure 4B), a mutant capable of binding to Dex, dimerising and translocating into the nucleus, but lacking transcriptional activity^30, 31^. These results show that Dex-induced sST2 production requires the participation of GRα through a transcriptional mechanism that might involve its binding to a site on any of the *IL1RL1* promoters.

### GC regulation of sST2 expression involves GR binding to functional GREs in the distal promoter

**Figure 3:**
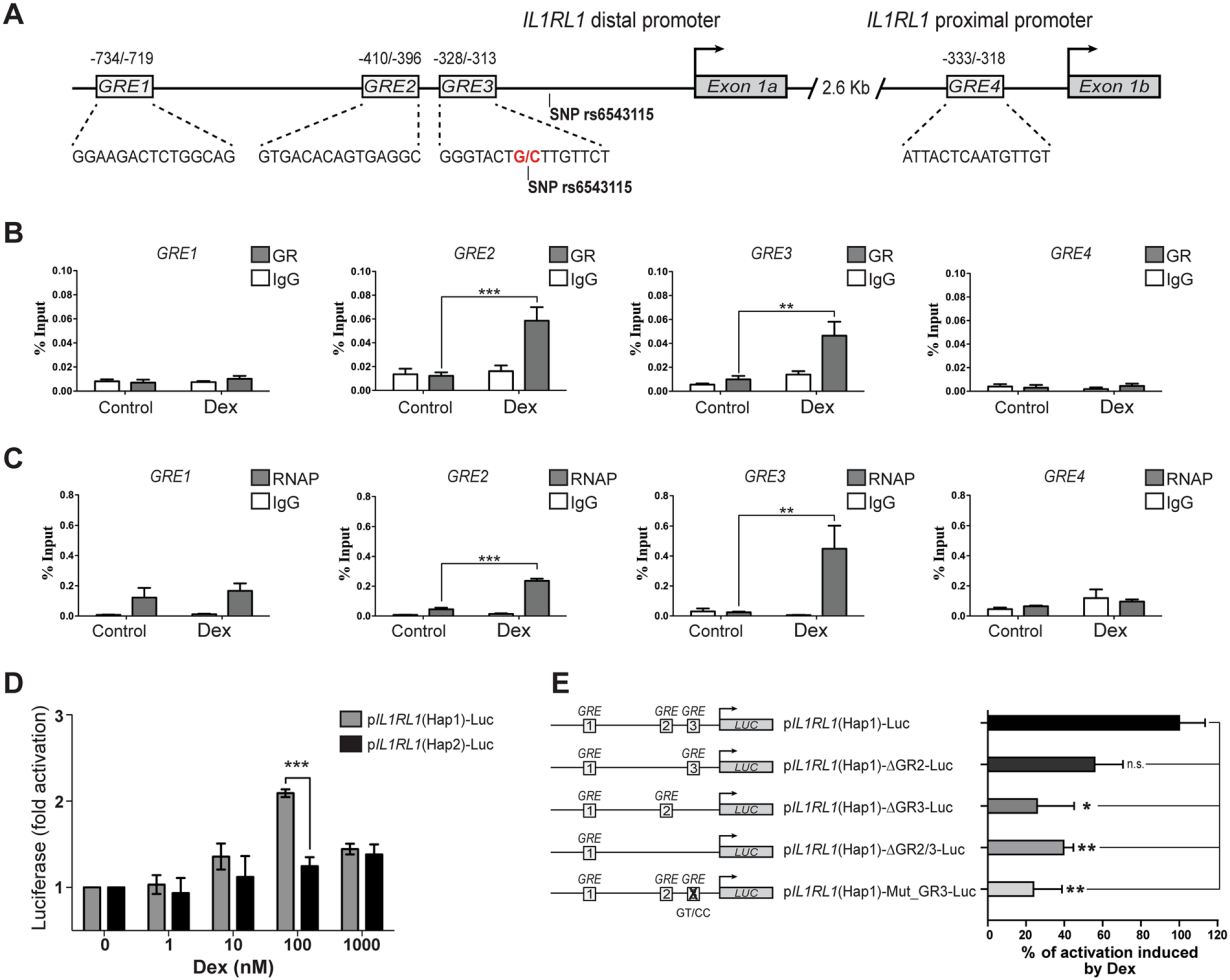
Dexamethasone enhances anti-GRα binding to the distal *IL1RL1* gene promoter in the rs6543115(C)/rs6543116(A) haplotype. (**A**) Schematic representation of GREs in the *IL1RL1* distal and proximal promoters, with the GRE position given in reference to Exon 1a (+1) for the distal promoter and Exon 1b (+1) for the proximal promoter. Boxes indicate the specific GRE sequence in each promoter. (**B**) ChIP assays for HMC-1 human mast cells stimulated with 100 nM Dex using an Ab57-specific polyclonal antibody against GRα, (**C**) an antibody against RNA pol II or control normal serum (IgG). The % input represents the % DNA precipitated using the specific antibody. n = 4. Kruskal-Wallis test with Dunn’s multiple comparison post-test. **p < 0.01; ***p < 0.001. (**D**) Luciferase activity associated with a 1211-bp region corresponding to the *IL1RL1* distal promoter containing the three putative GREs (GREs 1, 2 and 3) in haplotypes rs6543115(C)/rs6543116(A) (p*IL1RL1*(Hap1)-Luc) or rs6543115(G)/rs6543116 (**G**) (p*IL1RL1*(Hap2)-Luc, as determined in A549 cells treated with 0–1000 nM Dex. (**E**) Schematic luciferase reporter plasmids generated from p*IL1RL1*(Hap1)-Luc and percentage of luciferase activity induced by 100 nM Dex. Luciferase activity was normalised to Renilla (F/R = firefly/Renilla activity) and related to the control. Error bars represent the means ± SEM of 3 experiments. Two-way ANOVA. **p < 0.01; ***p < 0.001.

Our *in silico* analysis identified four putative GREs: three in the distal and one in the proximal human *IL1RL1* promoter, located 1.2 kb upstream of the transcription start site in exon 1a and 1 kb upstream of exon 1b, respectively (Fig. 3A and Supplementary material and methods). Alignments of putative GREs sequences identified, according to GRE consensus, predicted that GRE3 and GRE4 are the most conserved (Supplementary Figure 5). ChIP assays using Dex-treated HMC-1 cells showed that GRα bound predominantly to GREs 2 and 3 in the distal promoter, with a significant enrichment over the unstimulated condition (*p* < 0.001 and < 0.01, respectively) (Fig. 3B). These GRE sequences were also recovered with the RNA Pol II antibody, revealing a greater occupancy at these sites (*p* < 0.001 and *p* < 0.01, respectively) (Fig. 3C). These results indicate that GRE2 and GRE3 sites are functionally active in response to Dex stimulation.

### Functional effects of IL1RL1 SNP rs6543115 on promoter activity

The SNP rs6543115(C) allele, which we localised to GRE3, was previously found to be associated with asthma and atopic dermatitis^7, 8^, in which the IL33/ST2 system has also been implicated. To test whether the presence of SNP rs6543115(C) in GRE3 had a functional effect on GC-dependent sST2 induction, luciferase activity of a 1211 bp region of the *IL1RL1* distal promoter, containing haplotype clones Hap1 (rs6543115C, rs6543116A) or Hap2 (rs6543115G, rs6543116G), respectively, was measured in A549 cells. p*IL1RL1*(Hap1)-Luc-transfected cells stimulated with Dex at a dose range of 1–1000 nM showed a 2.0-fold increase in luciferase activity at 100 nM Dex compared with unstimulated cells (*p* < 0.001), whereas Dex induced only a slight increase in luciferase expression (1.32-fold) in p*IL1RL1*(Hap2)-Luc-transfected cells exposed to 1000 nM Dex as compared to controls (Fig. 3D). RU-486 pretreatment of p*IL1RL1*(Hap1)-Luc-transfected cells or of MMTV promoter-driven luciferase construct-expressing cells significantly reversed Dex-induced transcriptional activity (*p* < 0.01 and < 0.001) (Supplementary Figure 6).

### Functional effect of GRE3 in p*IL1RL1*(Hap1)-Luc in response to Dex

The proximity between GRE2 and GRE3 sites does not allow discrimination of their independent effects analysed through ChIP assays. To evaluate the contribution of both GRE2 and GRE3 on Dex-induced transcriptional activity of p*IL1RL1*(Hap1)-Luc we performed a mutational analysis generating the following deletion constructs: p*IL1RL1*(Hap1)-ΔGRE2-Luc, p*IL1RL1*(Hap1)-ΔGRE3-Luc and p*IL1RL1*(Hap1)-ΔGRE2/3-Luc and a site-directed mutagenesis GT > CC in the more conserved position of the half GRE3 site (p*IL1RL1*(Hap1)-Mut_GRE3-Luc). The luciferase activity in p*IL1RL1*(Hap1)-Luc induced by Dex was significantly decreased by 60 to 75% in A549 transfected cells with p*IL1RL1*(Hap1)-ΔGRE2/3-Luc and p*IL1RL1*(Hap1)-ΔGRE3-Luc mutants, respectively (Fig. 3E). No significant reduction was observed in p*IL1RL1*(Hap1)-ΔGRE2-Luc (Fig. 3E). Additionally, the luciferase activity in (p*IL1RL1*(Hap1)-Mut_GRE3-Luc) induced by Dex was decreased by 75% (p < 0.01) demonstrating the functionality of this GRE3 in response to Dex (Fig. 3E).

### Association of IL1RL1 SNPs rs6543115(C) and rs6543116(A) with UC and CD

The presence of SNPs rs6543115 G/C and rs6543116 G/A in our cohort was determined as the minor allele frequency (MAF), which was 0.46 in UC and CD patients and 0.37 in the control group; (Supplementary Table I). SNP rs76565432, located 8 nucleotides from rs6543115 and also identified by sequence analysis, had an MAF of 0.1 and showed no association with any group of patients. The distribution of the alleles and genotypes for SNPs rs6543115(C) and rs6543116(A) differed between the UC patients and control group (Table 1) and between the CD and control groups (Supplementary Table II). These alleles, as well as the homozygote genotype CC;AA (Hap 1), were associated with UC and CD susceptibility in a recessive model (Table 1 and Supplementary Table II, respectively).

**Table 1:** Distribution of genotypic and allelic frequencies of *IL1RL1* SNPs in UC patients and controls.

Genotype	Control (n = 137)	UC (n = 122)	*P* value	OR [95% CI]
GG;GG (Hap 2 homozygote)	53 (38.7%)	35 (28.7%)	0.017^a^	2.232 [1.159 to 4.300]
GC;GA (Heterozygote)	66 (48.2%)	62 (50.9%)	0.023^b^	2.076 [1.103 to 3.908]
CC;AA (Hap 1 homozygote)	18 (13.1%)	25 (20.4%)		
GG;GC/GG;GA	119 (86.9%)	97 (79.6%)	0.024^c^	2.005 [1.107 to 3.630]
CC;AA	18 (13.1%)	25 (20.4%)		
Haplotype alleles				
G;G (Hap 2)	172 (62.8%)	132 (54.1%)	0.025	1.450 [1.057 to 1.990]
C;A (Hap 1)	102 (37.2%)	112 (45.9%)		

^a^UC vs. control, genotype CC;AA vs GG;GG. ^b^UC vs. control, genotype CC;AA vs GC;GA. ^c^UC vs. control, genotype CC;AA vs GG;GC/GG;GA (recessive model). Fisher’s exact test OR (95% confidence interval). P values of <0.05 were considered significant.

### Association of IL1RL1 genotype with sST2 levels in UC patients

**Figure 4:**
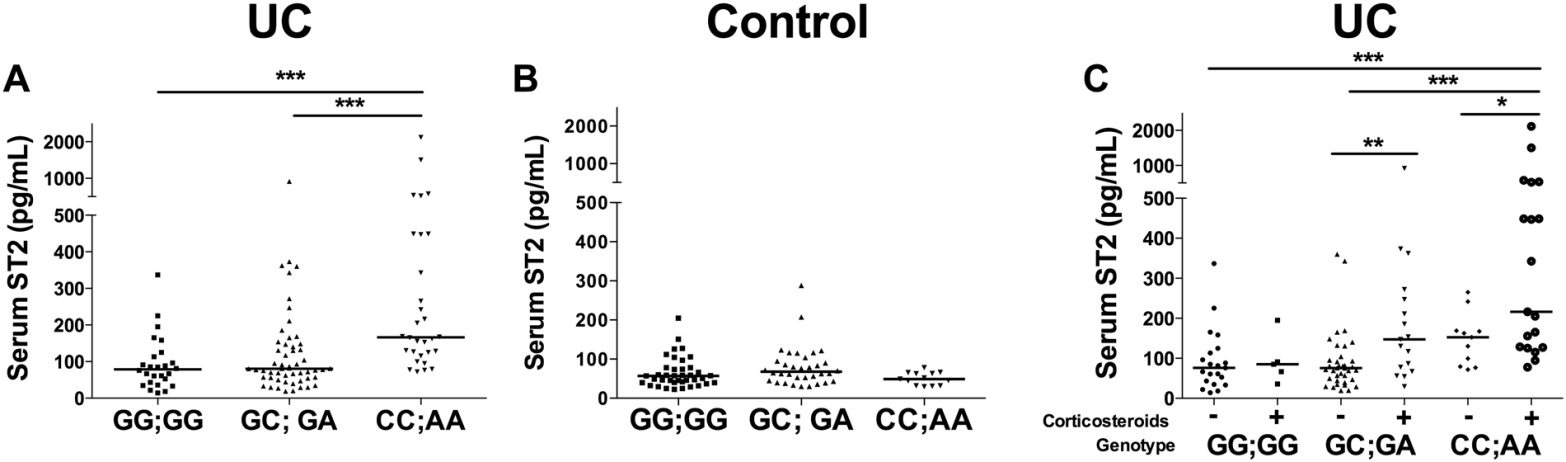
Corticosteroid treatment increases sST2 levels in UC patients with *IL1RL1* genetic variants. Serum ST2 levels determined by ELISA in genotyped UC patients (**A**), controls and (**B**) UC patients receiving or not receiving corticosteroids (**C**). Kruskal-Wallis test with Dunn’s multiple comparison post-test. *p < 0.05; **p < 0.01; ***p < 0.001.

Elevated sST2 levels in inflammatory conditions have been associated with the presence of SNPs in the *IL1RL1* distal promoter^7^. As we found an association between SNPs with the diseases, we wanted to further evaluate if there was a relation with corticosteroid treatment, as we found that GC induced sST2 in *ex vivo* organ mucosa explants as well as *in vitro* in HMC-1 cells. We found an association between corticosteroid treatment and UC patients harbouring the CC;AA genotype (OR = 3.06; 1.76–7.98; *p* = 0.036), suggesting a relationship between this genotype and serum sST2 levels in patients under GC treatment. Further analyses revealed significantly higher serum sST2 levels in UC patients with the CC;AA genotype as compared with the other genotypes (*p* < 0.01 vs. GG;GG *p* < 0.001 vs. GC;GA) (Fig. 4A), while in healthy control, sST2 levels were similar among the different genotypes (Fig. 4B). Moreover, UC patients harbouring the CC;AA or GC;GA genotype and receiving corticosteroids showed increased levels of sST2 (*p* < 0.05 and < 0.01, respectively) (Fig. 4C), whereas sST2 levels did not change in UC patients with the GG;GG genotype regardless of the use of corticosteroids. In CD patients, the CC;AA genotype showed higher serum sST2 levels compared with other genotypes, but differences were significant only in patients carrying the CC;AA genotype and receiving corticosteroid treatment (Supplementary Figure 7). Together, these results suggest that the CC;AA genotype and corticosteroid treatment contribute to the enhanced sST2 levels in UC patients and genetic susceptibility to the disease.

### GC-mediated induction of ST2 variants by leukocytes harbouring the CC;AA genotype

**Figure 5:**
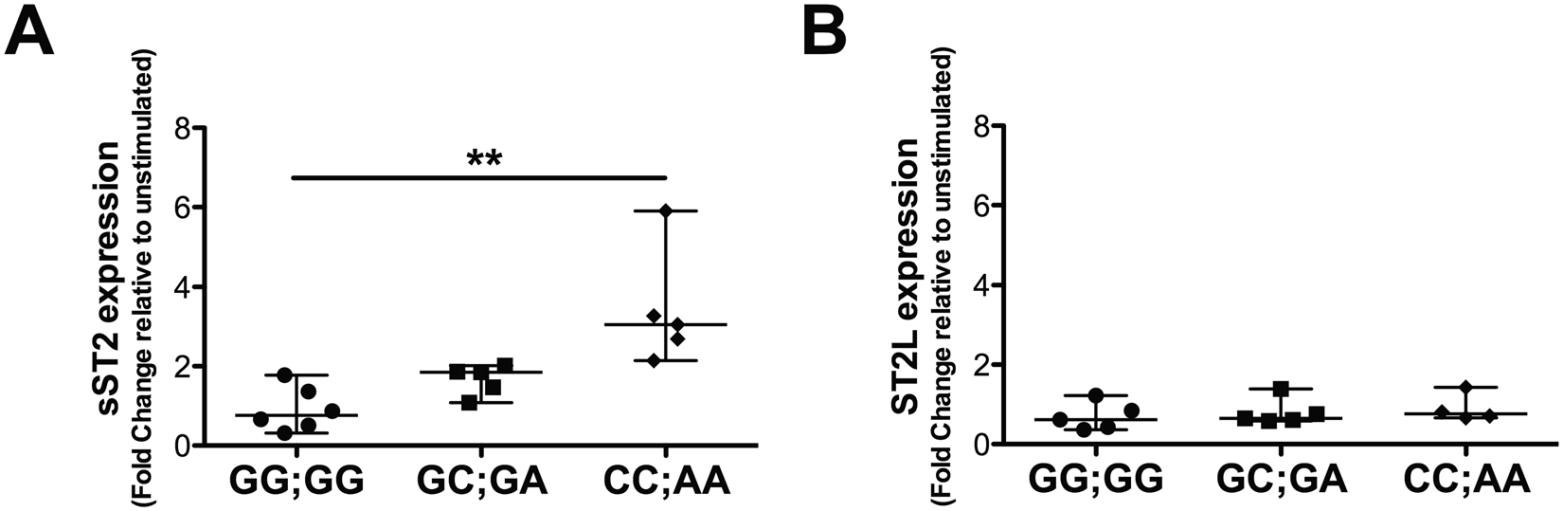
Dexamethasone increases sST2 transcript levels in peripheral leukocytes of healthy donors harbouring the CC;AA genotype. sST2 (**A**) and ST2L (**B**) transcripts in peripheral leukocytes of healthy donors harbouring the GG;GG (n = 5), GC;GA (n = 5) and CC;AA genotype (n = 5) and stimulated or not with 100 nM Dex for 6 hours were quantified by real-time PCR. mRNA content was normalised to 18 S rRNA and compared with the unstimulated. Kruskal-Wallis test with Dunn’s multiple comparison post-test. *p < 0.05.

Using another strategy to test whether the different genotypes influence the expression of ST2 variants induced by corticosteroids, we examined peripheral blood leukocytes derived from 15 healthy donors (female 12, male 3) and treated with Dex for differences among the three genotypes that would not be influenced by disease activity. The induction of sST2 after Dex treatment for the carriers of the CC;AA genotype (median 2.69; range 2.14–3.27) was greater than that in those with the GG;GG (median 0.87; range 0.51–1.78) or GC;GA (median 1.47; range 1.08–1.85) genotype (*p* = 0.050) (Fig. 5A), whereas ST2L expression was not altered in any of the genotype groups after Dex stimulation (*p* > 0.05) (Fig. 5B).

## Discussion

The main therapeutic goal in IBD is clinical remission, although efficacy of the used drug for treatment is evaluated based on activity score changes. Drugs currently used, such as corticosteroids and immunosuppressants, modify cellular and/or molecular responses in the intestinal mucosa; however, the exact mechanism(s) underlying their therapeutic effect remains unclear. Corticosteroids are currently the first-line treatment in patients with moderate-to-severe UC crisis^27^. Similar to cortisol, corticosteroid treatment down-regulates inflammatory responses via GR involvement, thus counteracting pro-inflammatory cytokine expression or inducing IκB. Here, we identify an additional mechanism by which corticosteroids exert their effects, involving sST2 overexpression detected in UC patients intestinal mucosa^14^. sST2 is a novel mediator that may reduce inflammatory processes driven by IL33 release from damaged epithelial cells in the intestinal mucosa of UC patients^32^. To counter the effect of IL33, mucosa resident and infiltrating mononuclear cells, granulocytes as well as mast cells may produce the decoy sST2 in the inflamed mucosa of UC patients. We and others have previously reported high sST2 levels in human intestinal biopsies^26, 33^. Our present analysis of *ex vivo* mucosa cultures treated with GCs supports the notion that cells present in the tissue respond through sST2 overexpression, an effect that might not involve post-translational modification, such as that seen in GC-mediated metalloproteinase-dependent ST2 ectodomain shedding, as shown for TLR2 soluble variant production^34^. Thus, sST2 variant generation likely involves transcriptional regulation mediated by glucocorticoid treatment.

Recently, the neutralising effect of sST2 on the IL33-induced signalling pathway has been demonstrated *in vivo* ^35, 36^, raising the possibility that sST2 is an activity biomarker for UC in light of the correlation of serum levels with ST2 intestinal content according to the activity score^14^. Furthermore, we found that sST2 reflected the inflammatory response and correlated with calprotectin levels in a one-year follow-up study^16^. The resulting increase in sST2 levels might be a compensatory mechanism for IL33-mediated inflammation, although it limits IL33-driven ST2^+^ T_Reg_ accumulation and function in the intestine^15^, thus perpetuating the inflammatory environment. Temporal information about sST2 production during chronic intestinal inflammation awaits further study.

We show here that GCs induce sST2 through a mechanism that regulates a GRE, designated GRE3, in the distal promoter of the *IL1RL1* gene harbouring the rs6543115(C) variant. GREs are composed of 15 nucleotides, containing two palindromic sequences separated by three nucleotides, which each homodimerize symmetrically with a GR molecule. GRE3 showed the highest identity to the consensus sequence (8/12 bases vs. 6/12 bases for GRE2) and its 3′ half was 100% identical to the consensus sequence, which might account for the Dex-induced functional activation of the *IL1RL1* distal promoter. While the proximity between GREs 2 and 3 (<80 nt, probably both GREs are compacted in the same nucleosome) could contribute to the activity observed for GRE2, RNA Pol II activity was detected mainly in GRE3 and moreover, the deletion and point mutation of this GRE significantly decrease the transcriptional activation induced by Dex. Furthermore, we demonstrate that a unique change in the GRE3 sequence enhances the transcriptional activity of the *IL1RL1* distal promoter and boosts GC-mediated sST2 production. The GR-mediated sST2 production in patients harbouring the rs6543115(C) variant may represent a mechanism involving higher receptor affinity for the GRE3 sequence. Together, these results suggest that GRE3 harbouring the rs6543115(C) variant is transcriptionally induced by Dex treatment through additive effects. Remarkably, the rs6543115(C) variant is located in the centre of the spacer sequence in GRE3; identity of the internal region affects GR binding, conformation and gene transcription^37^. Accordingly, analysis of the GC-responsive genes Tat (transactivator of transcription) and Sgk (serum/GC-regulated kinase 1), which have similar GRE palindromic sequences but different spacer regions, showed that the CCC versus TTT spacer sequence had a 4-fold increase in Dex-induced GR affinity^37^. Indeed, we found that a variation of G > C in the GRE3 spacer region of the *IL1RL1* distal promoter resulted in a 2-fold induction of Dex-mediated transcription. Although additional studies are required to explain how this variant could alter sST2 production in response to corticosteroid therapy.

IBDs are a group of multifactorial disorders that include a number of gene variants conferring genetic susceptibility. Polymorphisms related to the innate immune response have been associated with UC and CD pathogenesis. Among these, *NOD2*, *IL10*, *IL23R* and *IL12B* confer susceptibility to the disease^38–41^. GWAS analysis in a locus on chromosome 2 harbouring IL1 receptor superfamily genes and containing the genetic variant rs917997 in IL18RAP showed an association with IBD susceptibility^5^, although the strong LD at this locus precluded the fine-mapping needed to identify a genetic variant of *IL1RL1* using GWAS assay alone. Additional association studies, such as those presented herein, are required to fully demonstrate the impact of *IL1RL1* in UC. *IL1RL1* as part of this gene cluster has emerged as a key regulator in many immune and inflammatory disorders, such as atopic dermatitis and asthma^7, 8^. Recently, the non-coding SNPs rs2058660, rs2310173 and rs13015714 in the locus containing *IL1RL1* and rs3939286 in the IL33 gene were associated with IBD^10^. While none of these variants have been functionally related to gene expression or to the ST2/IL33 signalling pathway, five missense variants of *IL1RL1* correlated with higher sST2 levels and IL33 responsiveness (via ST2L) in cardiac diseases^9^.

We show here that SNP rs6543115 G/C located in GRE3 not only confers susceptibility to UC, but also accounts for corticosteroid regulation of sST2 by peripheral white blood cells. Previous studies have revealed that SNP rs6543115 G/C is associated with increased susceptibility to asthma and atopic dermatitis in different populations^7–9^.

In recent years, several reports have focused on the relationship between the ST2/IL33 signalling pathway and the immune response in IBD, particularly in UC. IL33 is a new member of the IL1 family and has been postulated to act as an alarmin^42^ released in response to tissue damage and activating inflammation^43^. To date, experimental animal models of IBD have been unable to demonstrate the role of IL33 in its etiopathogenesis^35^. Although ST2/IL33 axis neutralisation might represent an effective therapeutic target to control the inflammation that characterises IBD, new findings suggest that sST2 perpetuates inflammatory responses by restraining IL33-driven ST2^+^ T_reg_ cell accumulation and function in the intestine^15^.

During UC crisis or reactivation, changes in the epithelial architecture, mucosal organisation and increased infiltration of inflammatory cells have been observed^27^. Mast cells have been associated with IBD pathogenesis^21, 44–46^, and recent experimental animal models have demonstrated their role in homeostasis of the innate immune response by improving the barrier functions of the intestinal mucosa^18, 20, 29, 47^. Although the chronicity of the inflammatory cycle has been attributed to direct interaction between mast cells with classical cellular components of the adaptive immunity, such as resident macrophages and dendritic cells^32, 48, 49^. Further studies of the factors involved in these humoral and cellular interactions are needed to understand the impact of treatment in mast cell-related diseases, either through their stabilization or by modulating ST2 signalling pathway via sST2 production, especially during active UC. Here, we found an unexpected increase in sST2 production induced by Dex in a mast cell line, suggesting the participation of another GC-mediated mechanism involving the production of an IL33 inhibitor that might affect the inflammatory process promoted by damaged intestinal mucosa.

**Figure 6:**
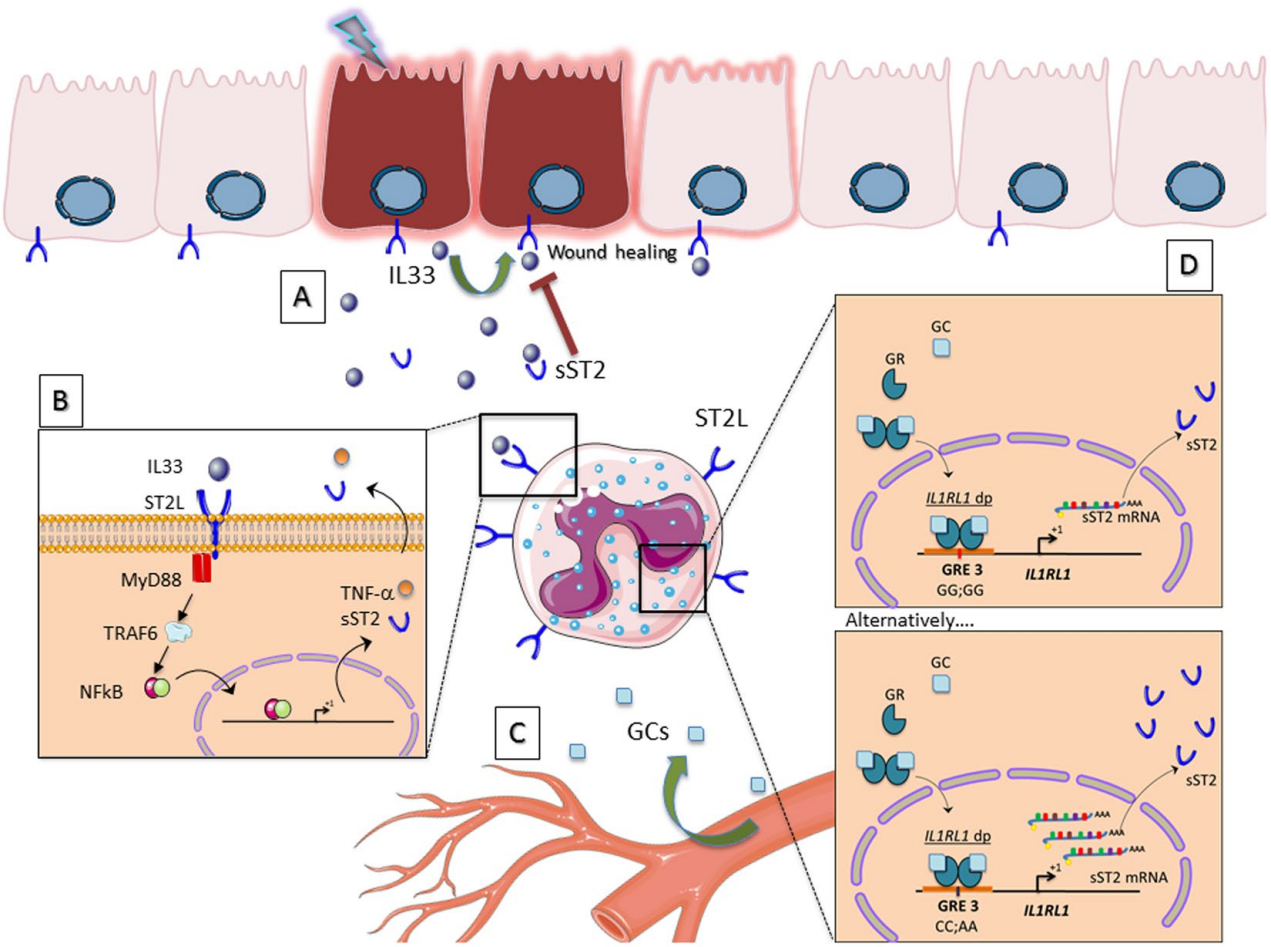
Model of sST2 expression regulated by GCs in cells of the intestinal mucosa. (**A**) The chronic inflammation observed in IBD patients promotes damage of the intestinal epithelium that results in IL33 release. This cytokine through its regenerative mechanisms could promote re-epithelization and mucosa healing. (**B**) On the other hand, through its pro-inflammatory effects, it mediates the production of other inflammatory mediators in a wide range of immune cells exacerbating the inflammatory response. (**C**) The administration of exogenous GCs can modulate the inflammation. (**D**) In subjects harbouring the CC;AA haplotype, which involves the GRE3 located in the distal promoter of the *IL1RL1* gene, a greater expression of sST2 can be induced by GCs in immune cells, which could dramatically limit the regenerative activity of IL33 by perpetuating the damage at the intestinal level.

In conclusion, we show that the genetic variant rs6543115(C) in the distal *IL1RL1* promoter confers susceptibility to UC as well as increased sST2 expression (see diagram in Fig. 6). Moreover, the rs6543115(C) variant and the corticosteroid treatment of peripheral white blood cells contributes to the enhanced sST2 levels through a mechanism involving the GRE3. Further studies with larger patient cohorts are needed to confirm that this genetic variant is relevant to both the pathology of UC and corticosteroid response. Identification of genetic variants in the IL33/ST2 signalling pathway will be important in facilitating the choice of appropriate therapeutic targets, especially for UC patients at risk of unfavourable disease evolution.

## Methods

### Study sample

This was a prospective and case-controlled observational study. Participants were recruited from the Gastroenterology Unit at Clinica Las Condes from 2011 to 2013. A total of 329 participants were included in the genetic analysis. Patients were grouped based on endoscopic and histological criteria: UC, n = 122; CD, n = 70 (IBD, n = 192); control group of healthy controls, n = 137.

For organ culture experiments, UC patients (n = 18) and control individuals (n = 10) were recruited from the Gastroenterology Unit at Clinica Las Condes and the endoscopy Department at the Royal London Hospital, UK. Patients and controls were included and grouped according to the corticosteroid used.

### Clinical Assessment

The patients were diagnosed and classified based on standard clinical, endoscopic and histological criteria. The clinical characteristics are summarised in Table 2.

**Table 2:** Clinical characteristics of participants included in the study.

	IBD	UC	CD	Healthy control
Number patients	192	122	70	137
Female/Male	110/82	73/49	44/26	76/61
Age at entry (Mean ± SE), years	38.81 ± 1.02	37.76 ± 1.12	40.75 ± 1.98	45.36 ± 1.83
Extension of disease				
Proctitis		26		
Left-sided colitis		30		
Extensive		66		
Location of CD				
Ileon			17	
Colon			34	
Ileocolonic			19	
Disease behavior, CD				
Inflammatory			62	
Stricturing			7	
Penetrating			1	
Severity of the previous episode^**a**^				
Inactive		30	15	
Mild		28	18	
Moderate		38	26	
Severe		26	11	
Medication at endoscopy^**b**^				
No medication	24	18	6	
5-ASA	125	86	39	
Corticosteroids	47	33	14	
Azathioprine	54	24	30	
Biologics (Infliximab)	17	4	13	
Intestinal resection surgery	32	16	16	

^a^Severity according Mayo endoscopic subscore in UC and according to SES in CD (0 = Inactive; 1 = Mild; 2 = Moderate; 3 = Severe). ^b^Some patients received combined therapies.

During the study process, all included UC and CD patients were subjected to colonoscopy, biopsies were taken from inflamed mucosa and immediately frozen in liquid nitrogen and stored at −80 °C until analysis or collected in 2% paraformaldehyde (PFA). A blood specimen (4 mL) was obtained from each patient for further molecular and biochemical analysis.

IBD patients were classified according to the clinical criteria of the Montreal classification^50^. Inclusion criteria for the study were: IBD diagnosed patients (UC or CD), >18 years. Exclusion criteria were: non-classifiable inflammatory disease, indeterminate colitis, infectious ileocolitis, asthma, atopic dermatitis, and history of autoimmune diseases, celiac disease and hypertension.

In UC and CD patients, tissue damage was determined using the endoscopic Mayo Subscore and the Simple Endoscopic Score for Crohn’s Disease (SES-CD), respectively. Each biopsy was graded on a scale of 0–3 (0 = normal; 1 = mild; 2 = moderate; 3 = severe, including those patients with active ulceration)^27^. Control samples were obtained from unaffected intestinal tissues of Sporadic colorectal cancer patients.

### Ethics Statement

All participants provided informed consent. The study was approved by the Institutional Review Board at Clinica Las Condes and performed according to human experimental guidelines and approved by the NRES Committee London City, and East and KCL Infectious Diseases BioBank, and Local Research Ethics Committee, as authorized by the Southampton and South West Hampshire Research Ethics Committee (ref 10/H0704/74). Clinical investigation was conducted according to Declaration of Helsinki principles, with participants identified only by number.

### Laboratory testing

The serum concentrations of sST2 were measured using an enzyme-linked immunosorbent assay (ELISA) kit for human ST2 (DuoSet, R&D Systems, Minneapolis, MN, USA) according to the manufacturer’s instructions. Blood samples, collected during colonoscopic procedure, were centrifuged and serum was stored at −80 °C. Serum samples were treated with protein A/G PLUS-Agarose (Santa Cruz Biotechnology, Santa Cruz, CA, USA). sST2 concentrations were measured using enzyme-linked immunosorbent assay (ELISA) kit for human ST2 (DuoSet, R&D Systems, Minneapolis, MN, USA) according to the manufacturer’s instructions, and expressed in pg/mL. The assay is stable over time with a detection limit of 20 pg/mL. Within-run and total coefficients of variation ranged between ≤ 2.5% and ≤ 4.0%, respectively.

### Genotyping

Genomic DNA was obtained from peripheral blood leukocytes and purified by erythrocyte lysis with ACK buffer (0.15 M NH_4_Cl, 10 mM K_2_HPO_4_, 0.1 mM EDTA). Leukocyte extracts were treated with proteinase K and subsequent extraction with basic phenol/chloroform/isoamyl alcohol (25:24:1). DNA was quantified by spectrophotometric analysis (Synergy 2, Biotek Instruments, Inc., Winooski, VT, USA) and used as template for PCR amplification and sequencing. A 569 bp region of *IL1RL1* distal promoter was amplified using primers F8 (5′-CCTGTCAGCTTCTGAGAATTGCGTG-3′) and R8 (5′-CCAGTTTATCAGTTAAGAGACAGGAA-3′). PCR products were visualized on 1% agarose gel, then purified using QIAquick gel extraction kit (Qiagen, Valencia, CA, USA) and sequenced with internal primers F9 (5′-GAAAGAAACACCAAATAAAGCAAC-3′) and R9 (5′-CCACATTCTCCCTATTTGAAAGATC-3′) using 3730XL DNA Sequencer (Macrogen Inc., Korea). Finally, SNPs were determined by analyzing the corresponding electropherograms with ChromasPro software v1.41 (Technelysium, Pty Ltd., Tewantin, QLD, Australia). Multiple alignments of the analyzed sequences were performed using Vector NTI Advance 10.0 software (IBI, New Haven, CT, USA).

### *In silico* analysis of GRE sites in the IL1RL1 promoters

Bioinformatics analysis of GRE sites in both distal and proximal promoters was performed using published human *IL1RL1* sequences from the GenBank™/ EMBL database (accession No. AC007248). The transcription factor binding site search was performed using TFsearch version 1.3 (Computational Biology Research Center, National Institute of Advanced Industrial Science and Technology, Tsukuba, Japan), MatInspector (Genomatrix Software, Munchen, Germany), TFblast, TFsitescan, P-Match, Signal Scan and The Jaspar database. The criteria for the selection of potential GRE sites consisted of sequence identification by at least 3 software algorithms with a score greater than 70%.

### Cell culture studies

#### Organ Cultures

Intestinal tissue explants (1 mm^3^ in size) were placed in 12-well culture plates (one explant per well) and cultured at 37 °C and 5% CO_2_ in 1 ml serum-free DMEM medium supplemented with 100 U/ml penicillin and 100 μg/ml streptomycin, as previously described. For corticosteroid effect experiments, dexamethasone, prednisone or prednisolone (Steraloids, Inc. Newport, RI, USA) was added to the culture medium at a concentration of 100 nM as previously described. For recombinant human IL-33 (rhIL-33, R&D Systems, Minneapolis, MN, USA) effect experiments, 50 ng/ml was added to the culture medium as reported in *in vitro* and *ex vivo* model^51, 52^. After 24 hours *ex vivo* culture, supernatants were collected and stored at −80 °C until used for ST2 and cytokine measurement by ELISA (DuoSet, R&D Systems, Minneapolis, MN, USA) as previously above.

#### Immunofluorescence

ST2 or ST2 tryptase double labelling was detected in 2% PFA-fixed, paraffin-embedded patient biopsies that were cut into 4-µm sections and subjected to ST2 tryptase double labelling immunofluorescence analysis. A mouse monoclonal antibody against human ST2 (MAB523, R&D Systems) and an Alexa 546-tagged secondary goat antibody against mouse IgG (Invitrogen/Life Technologies, Carlsbad, CA, USA) were used. Next, the samples were blocked again and incubated with a mouse monoclonal antibody against human tryptase (M7052, Dako, Glostrup, Denmark) followed by an Alexa 488-tagged secondary antibody against mouse IgG (Invitrogen). Nuclei were stained using DAPI (Thermo/Life Technologies, Carlsbad, CA, USA). Images were captured using an Olympus Confocal Laser Scanning Biological Microscope FV10i (Olympus America Inc., Melville, NY, USA) and processed using ImageJ (NIH, USA). Negative controls were prepared under conditions identical to those described above by replacing the primary antibodies with an isotype-identical serum.

#### Cell culture and reagents

The human mast cell line HMC-1 and the human alveolar basal epithelial cell line A549, derived from a human mastocytoma and from an adenocarcinoma, respectively, were used for the *in vitro* assays. The human mast cells HMC-1 and the human alveolar basal epithelial cells A549, were grown in Iscove’s Modified Dulbecco Medium and Dulbecco’s modified Eagle’s medium (IMDM and DMEM Gibco-Life Technologies, NY, USA), respectively. Culture media were supplemented with 5% fetal bovine serum (FBS), 25 mM Hepes, 35 µM sodium bicarbonate, 100 UI/mL penicillin/streptomycin. HMC-1 cells were cultured at 1 × 10^6^ cell/mL in IMDM FBS free for 18 hours before stimulation.

#### Lentivirus preparation

A wild type version of the human GC receptor alpha gene (hGRα) and a dominant mutant (hGRα-Δ428–490) were cloned into a lentiviral expression system to produce vectors for transgenesis^53^. 293 T cells were cotransfected with lentiviral constructs, VSVg-encoding plasmids and helper plasmids. After 16 hours, the medium was replaced, and at 60 hours post-transfection, the vectors were harvested and centrifuged to remove cellular debris. Then, aprotinin was added to a final concentration of 1 μg/mL. For transduction, the target cells were preincubated with 6 μg/mL polybrene (Sigma–Aldrich, St. Louis, MO, USA) for 30 minutes, and the vectors were added at a dilution of 50% in medium. After transduction (72 hours), the cells were divided for confirmation of expression (immunofluorescence) and phenotype testing.

### ST2 promoter construct and reporter gene assay

A region of the distal promoter sequence of 1211 bp was cloned into the pGL3 basic vector (Promega Corporation, Madison, WI, USA), including a portion of *IL1RL1* exon 1a between KpnI and XhoI restriction sites. The two SNPs described in this region (rs6543115 G/C and rs6543116 G/A)^7^ were in complete LD. Two haplotype clones were generated and verified by direct sequencing (Macrogen Inc., Seoul, Korea). We transfected the Hap1 and Hap2 reporter plasmids pGL3-basic (p*IL1RL1*(Hap1)-Luc and p*IL1RL1*(Hap2)-Luc) into A549 cells because these cells, unlike HMC-1 cells, display dish adherence and higher transfection efficiency. Additionally, site-directed mutagenesis was performed in p*IL1RL1*(Hap1)-Luc using the Quick-Change site-directed mutagenesis kit (Stratagene; La Jolla, CA) according the manufacturer’s protocol. Four constructions were generated: p*IL1RL1*(Hap1)-ΔGRE2-Luc (deletion between positions 1543 to 1557); p*IL1RL1*(Hap1)-ΔGRE3-Luc (deletion between positions 1462 to 1476); p*IL1RL1*(Hap1)-ΔGRE2/3-Luc (deletion between positions 1462 to 1557) and p*IL1RL1*(Hap1)-mutGRE3-Luc (positions 1553–1554 sequence GT in GRE-half site was mutated to CC). As an internal control for the transfection efficiency, A549 cells were transfected with pGL3-hRL (Renilla luciferase vector; Promega, Madison, WI, USA) and 24 hours later, the cells were stimulated with a range of concentrations of dexamethasone (Steraloids, Inc. Newport, RI, USA). Transit-LT1 was used as the transfection reagent (Mirus Bio LLC, Madison, WI, USA). The pGL3-MMTV plasmid (plasmid containing GREs from a mouse mammary tumour virus) (Promega, Madison, WI, USA) was used as a GC-dependent response control. After 18 hours, luciferase activity was measured using a Dual Luciferase Reporter Assay Kit (Promega, Madison, WI, USA).

### Chromatin immunoprecipitation assays

ChIP assays were performed as previously described, with modifications^54^. Briefly, HMC-1 mast cells stimulated with or without Dex for 2 hours were collected in 1% PBS, formaldehyde cross-linked for 15 minutes, and then lysed in a mixture containing 25 mM Tris-HCl [pH 8.0], 5 mM MgCl_2_, 10 mM EDTA, 1% SDS, 1% Triton X-100, 162.5 mM NaCl, 25 µM MG-132 and a complete protease inhibitor cocktail. DNA fragments with an average size of 0.2 to 0.5 kb were obtained. Immunoprecipitation was performed with polyclonal antibodies specific to GR using a previously characterised antibody against the human GC receptor Ab57^55^ and RNA polymerase II (Santa Cruz Biotechnology, CA, USA) or isotype-matched immunoglobulin G (IgG; Abcam, CA, USA) as a control. Immunocomplexes were collected followed by the recovery of DNA. Instead of the elution step (1% SDS and 100 mM Na_2_HCO_3_) after washing, the immunocomplexes were eluted in 10 mM DTT. The supernatant was further diluted 1:40 in ChIP dilution buffer (0.01% SDS, 1.1% Triton X-100, 1.2 mM EDTA, 167 mM Tris-HCl [pH 8.1], and 167 mM NaCl). Aliquots of each recovered DNA sample were assayed by quantitative PCR (qPCR) to detect the distal *IL1RL1* promoter region spanning bp -1000 to -100 downstream of the transcriptional start site and the proximal promoter region spanning bp −600 to −200. The following oligonucleotides were used: GRE1 distal promoter (fwd 5′-GAGAGAGAGGTTAGAGAATTTGCGC-3′, rev 5′-ATCAGGATGTGCCATCTGCC-3′); GRE2 (fwd 5′-AGCACACGAGATGTGTCAAAG-3′, rev 5′-CAAAGCCTCACTGTGTCACC-3′); GRE3 (fwd 5′-CACCAAATAAGCAACTTGCTG-3′, rev 5′-AGCAAAACCTCCCTAACACC-3′); GRE4 proximal promoter (fwd 5′-GAGACACTCCTCCCATCCTGA-3′, rev 5′-CGGGGTACAGGCAATAAGCAT-3′). The samples were normalised to the initial input and expressed as the percentage of chromatin pull-down (compared with the input).

### Real-time PCR

HMC-1 cells were stimulated with 1–1000 nM of the synthetic GC dexamethasone (Steraloids, Inc. Newport, RI, USA) for 8 hours, and the expression analyses were performed using real-time qPCR (RT-qPCR). Total RNA from each sample was extracted with TRIzol® (Invitrogen/Life Technologies, Carlsbad, CA, USA) following the manufacturer’s protocol, integrity was analyzed by electrophoresis in 1% agarose gel and concentration was determined by spectrophotometric analysis (Synergy 2, Biotek Instruments, Inc., Winooski, VT, USA). Then, two μg of RNA was used to synthesize cDNA using oligo-dT (Promega, Madison, WI, USA) and RT-affinity (Agilent Technologies Inc., CA, USA) in a final volume of 20 μL. All mRNAs expression analyses were performed by real-time qPCR (RT-qPCR) using the Brilliant® II kit SYBR® Green QPCR Master Mix (Agilent Technologies Inc., CA, USA) and sST2 primers (fwd 5′-GGCACACCGTAAGACTAAGTAG-3′; rev: 5′-CAATTTAAGCAGCAGAGAAGCTCC-3′), ST2L (fwd 5′-ATGTTCTGGATTGAGGCCAC-3′, rev: 5′-GACTACATCTTCTCCAGGTAGCAT-3′), MKP-1 (fwd 5′-CCTGACAGCGCGGAATCT-3′, rev: 5′-GTGATACGCACTGCCCAGGTA-3′) or 18 S rRNA (fwd 5′-GTGGAGCGATTTGTCTGGTT-3′; rev: 5′-CGCTGAGCCAGTCAGTGTAG-3′) at a final concentration of 250 nM in a final volume of 20 μL. Amplification was performed with Mx3000 P QPCR System (Agilent Technologies Inc., CA, USA). As internal control amplification, 18 S rRNA was used and relative transcript quantification was performed by the 2^−ΔΔCT^ method.

### Flow cytometry analysis

HMC-1 cells stimulated with 100 nM Dex (Steraloids, Inc. Newport, RI, USA) for 24 and 48 hours, were harvested and stained with mouse Anti-Human ST2/IL-1 R4 PE-conjugated Monoclonal Antibody (R&D Systems, Minneapolis, MN, USA) or isotype control antibody for 30 min at 4 °C. Relative mean fluorescence intensity (MFI) in the samples was measured using the FACSCalibur flow cytometer (BD Biosciences) and analysed by FlowJo software (FlowJo LLC, Ashland, OR, USA).

### ST2 variants production by white blood cells carrying the CC;AA genotype

Venous blood was collected from 15 healthy volunteers with three genotypes (n = 5 for each one: CC;AA, GC;GA, GG;GG) into heparinized (10 U/ml) syringes. Dexamethasone was added to blood at a final concentration of 10^−7^ M (diluted in PBS) and at cultured at 37 °C and 5% CO_2_ for 6 hours^56^. Leukocytes were collected after lysis from blood incubated with the vehicle (PBS) or blood stimulated with 10^−7^ M Dex. Blood was centrifuged at 1,000 x *g* for 5 min, and the packed cell pellet was lysed twice with 2 vol of ACK (Ammonium-Chloride-Potassium) Lysing Buffer (Invitrogen). White blood cells were washed with PBS and processed for RNA isolation using RNeasy Mini Kit spin columns (Qiagen, USA).

The same protocols and primers detailed above for RNA from HMC-1 cells were used. For each sample, the mRNA abundance was normalized to the amount of 18 S rRNA. Data analysis was performed using the ΔΔCt method and results were expressed in relative mRNA levels. Expression levels were compared among three groups by the non-parametric test.

### Statistical analyses

Case-control analyses were performed using the χ^2^ or Fisher exact test. The associations between *IL1RL1* genetic variants and the phenotypic characteristics of UC and CD were estimated based on the odds ratio (OR) with 95% confidence intervals (CIs) using logistic regression analysis. Statistical analysis of the data was performed using t-test, two-way ANOVA, or for non-parametric distribution Mann-Whitney and Kruskal-Wallis tests, with Dunn’s multiple comparison post-test when was appropriated. For each statistical test, a 2-tailed *p* values < 0.05 were considered significant.

## Electronic supplementary material

Supplemental Information
