# Supplementary material for: A functional IL1RL1 variant regulates corticosteroid-induced sST2 expression in ulcerative colitis
Source: Sci Rep. 2017 Aug 31;7:10180. doi: 10.1038/s41598-017-10465-0 (PMC5579262; doi:10.1038/s41598-017-10465-0)

# Supplemental Information

**A functional *IL1RL1* variant regulates corticosteroid-induced sST2 expression in ulcerative colitis.**

David Díaz-Jiménez[1,2], Lucía Núñez[1], Marjorie De la Fuente[1,3], Karen Dubois-Camacho[1], Hugo Sepúlveda[4], Martín Montecino[4], Alejandro Torres-Riquelme[1], Paulina García-González[1], Jonás Chnaiderman[5], Anna Vossenkamper[6], Thomas T. MacDonald[6], Daniela Simian[3], María-Julieta González[7], John A. Cidlowski[2], Rodrigo Quera[8#], Marcela A. Hermoso[1#]

**Suplemental Data – Legends**

**Supplementary Figure 1:** Corticosteroid treatment increases serum levels of ST2 in CD. Serum ST2 levels in CD patients grouped according to therapies (A) or according to disease activity and corticosteroid (CORT) treatment (B) were determined by ELISA. 5-ASA (5-aminosalicylic acid), AZA (azathioprine), corticosteroids (hydrocortisone, prednisone and prednisolone), IFX (infliximab). Differences were assessed using the Kruskal-Wallis test and Dunn's multiple comparison post-test. *$p$<0.05; **$p$<0.01.

**Supplementary Figure 2:** Effect of corticosteroids and IL33 on sST2 and IL6 expression. Levels of sST2 (A), IL6 (B) and IL33 (C) in conditioned media of biopsies from healthy controls (HC) or UC patients, stimulated or not with 100 nM prednisone ("a") or prednisolone ("b"), and levels of sST2 (D) and IL6 levels (E) in conditioned media of biopsies, incubated or not with rh IL33 (50 ng/ml), were determined by ELISA. Differences between medians were assessed using the Mann-Whitney U-test. *$P < 0.05$; **$P < 0.01$.

**Supplementary Figure 3:** Immunofluorescence detection of ST2 in mast cells from UC and control intestinal mucosal sections. Mouse anti-human ST2 antibody and goat anti-rabbit antibody conjugated to Alexa Fluor 594 (red) and mouse anti-human tryptase antibody and a goat anti-rabbit antibody conjugated to Alexa Fluor 488 (green) were used for indirect immunofluorescence assays. DNA was counterstained with DAPI (blue). Representative photomicrographs: n=3. White arrows: infiltrating cells. Inset: magnification of infiltrating cells. Scale bar: 70 mm.

**Supplementary Figure 4:** Dexamethasone-induced MKP-1 expression in mast cells. (A) Production of MKP-1 mRNA in HMC-1 cells after treatment for 6 hours with 0-1000 nM Dex, as detected by qPCR (mRNA content normalised to 18S rRNA). n=5. (B) MKP-1 mRNA expression in HMC-1 cells transduced with lentiviral vectors carrying a mutant hGRα (hGRα-Δ428-490), hGRα, an irrelevant vector (IV), or non-transduced (mock) and treated with 100 nM Dex (mRNA content normalised to 18S rRNA and compared with the control). Error bars represent the means ± SEM of 4 independent experiments. The Kruskal-Wallis test with Dunn's multiple comparison post-test was used for each analysis. *$P < 0.05$; **$P < 0.01$.

**Supplementary Figure 5:**

Schematic representation of putatives GREs present in the distal and proximal promoter of *IL1RL1* gene and multiple alignment motif tree using Stamp. (B) Luciferase activity in A549 cells expressing a p*IL1RL1*(Hap1)-Luc, p*IL1RL1*(Hap2)-Luc or pEmpty-Luc and (C) mouse mammary tumour virus (MMTV) promoter-driven luciferase construct, stimulated with 100 nM Dex and pretreated with 1 mM of the receptor antagonist (RU-486). Error bars represent the means ± SEM of 3 experiments. Two-way ANOVA. **$p<0.01$; ***$p<0.001$.

**Supplementary Figure 6:** Corticosteroid treatment increases sST2 levels in CD patients with *IL1RL1* genetic variants based on genotyping. Serum ST2 levels determined by ELISA in genotyped CD patients receiving or not receiving corticosteroids. Kruskal-Wallis test with Dunn's multiple comparison post-test. *$P < 0.05$; **$P < 0.01$; ***$P < 0.001$.

**Suplemental Data – Tables**

**Supplemental Table I**: Minor allele frequencies of IL1RL1 SNPs in UC and CD patients

| SNP | Minor Allele | UC (n=122) MAF* *P value* *OR [95% CI]* | CD (n=70) MAF *P value* *OR [95% CI]* | Control (n=137) MAF |
|---|---|---|---|---|
| **rs6543115** | C | 0.46 0.0003<br>1.93 [1.34-2.72] | 0.46 0.073<br>1.46 [0.97-2.21] | 0.37 |
| **rs76565432** | G | 0.05 0.3594<br>0.69 [0.32-1.46] | 0.09 0.438<br>1.37 [0.65-2.87] | 0.07 |
| **rs6543116** | A | 0.46 0.0003<br>1.93 [1.34-2.72] | 0.46 0.073<br>1.46 [0.97-2.21] | 0.37 |

* Minor Allele Frequency
Odds Ratio (OR) = 95% confidence interval
Fisher´s exact test

**Supplemental Table II**: Distribution of genotypic and allelic frequencies of IL1RL1 SNPs in CD patients

| Genotype | Control (n=137) | CD (n=70) | *P* value | OR [95% CI] |
|---|---|---|---|---|
| **GG; GG (Hap 2 homozygote)** | 53 (38.7%) | 27 (38.6%) | 0.0028[a] | 3.368 [1.518 to 7.473] |
| **GC; GA (Heterozygote)** | 66 (48.2%) | 21 (30.0%) | 0.0004[b] | 4.400 [1.935 to 10.01] |
| **CC; AA (Hap 1 homozygote)** | 18 (13.1%) | 22 (31.4%) | | |
| **GG;GC/GG;GA** | 119 (86.9%) | 48 (68.6%) | 0.0004[c] | 3.819 [1.830 to 7.974] |
| **CC;AA** | 18 (13.1%) | 22 (31.4%) | | |
| **Haplotypes alelles** | | | | |
| **G;G** | 172 (62.8%) | 75 (53.6%) | 0.0103 | 1.743 [1.151 to 2.639] |
| **C;A** | 102 (37.2%) | 65 (46.4%) | | |

[a] CD vs. control, genotype CC;AA vs GG;GG;
[b] CD vs. control, genotype CC;AA vs GC;GA
[c] CD vs. control, genotype CC;AA vs GG;GC/GG;GA (recessive model)
Fisher´s exact test with Yates' correction OR (95% confidence interval)
*P* values of <0.05 were considered significant

## Suplemental Data – Figures

## Supplementary Figure 1

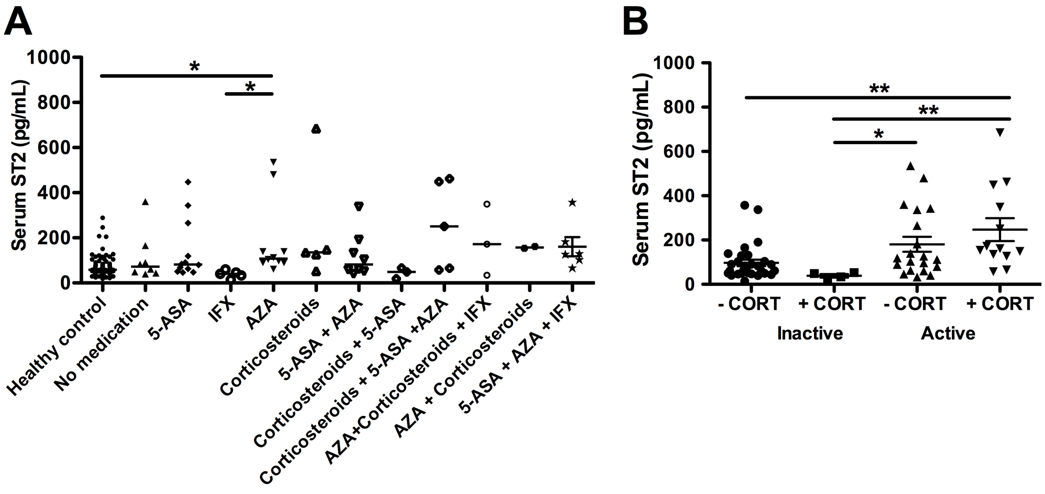


## Supplementary Figure 2

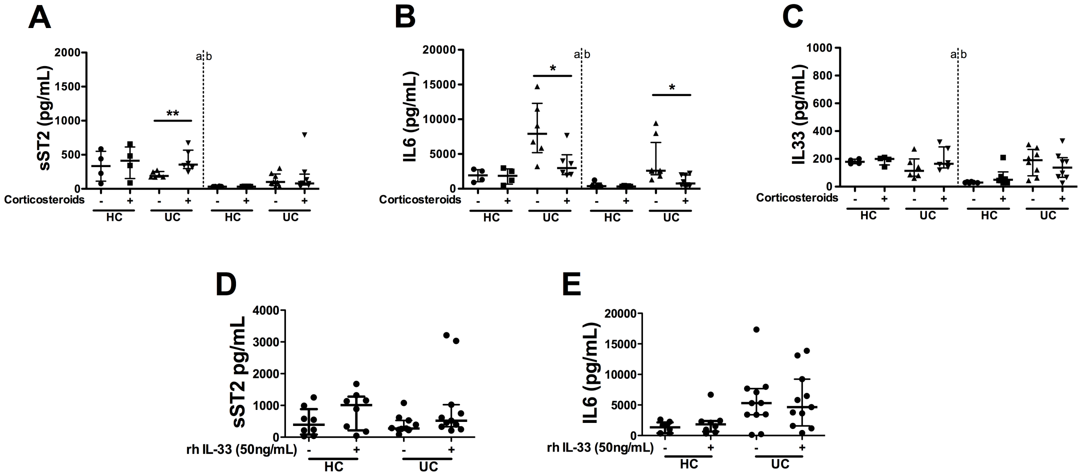

## Supplementary Figure 3

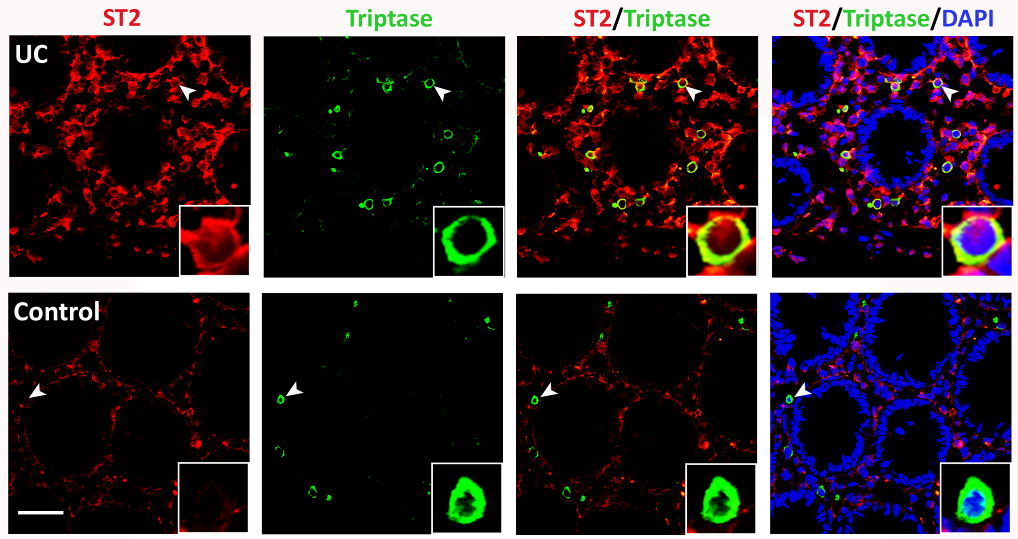


## Supplementary Figure 4

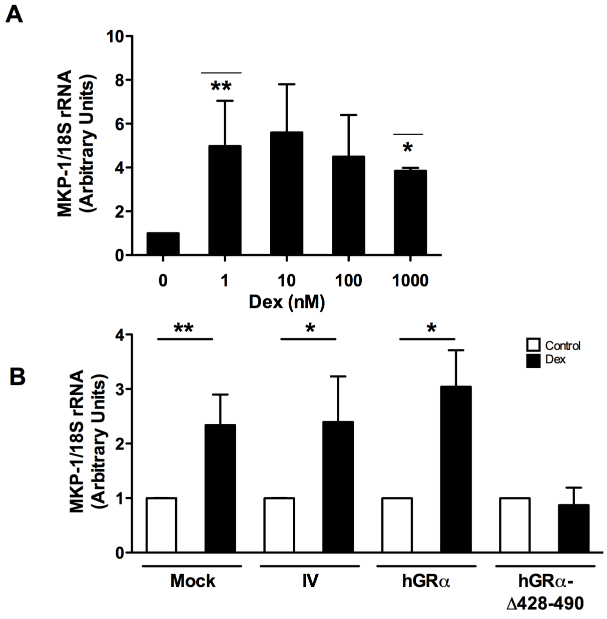

## Supplementary Figure 5

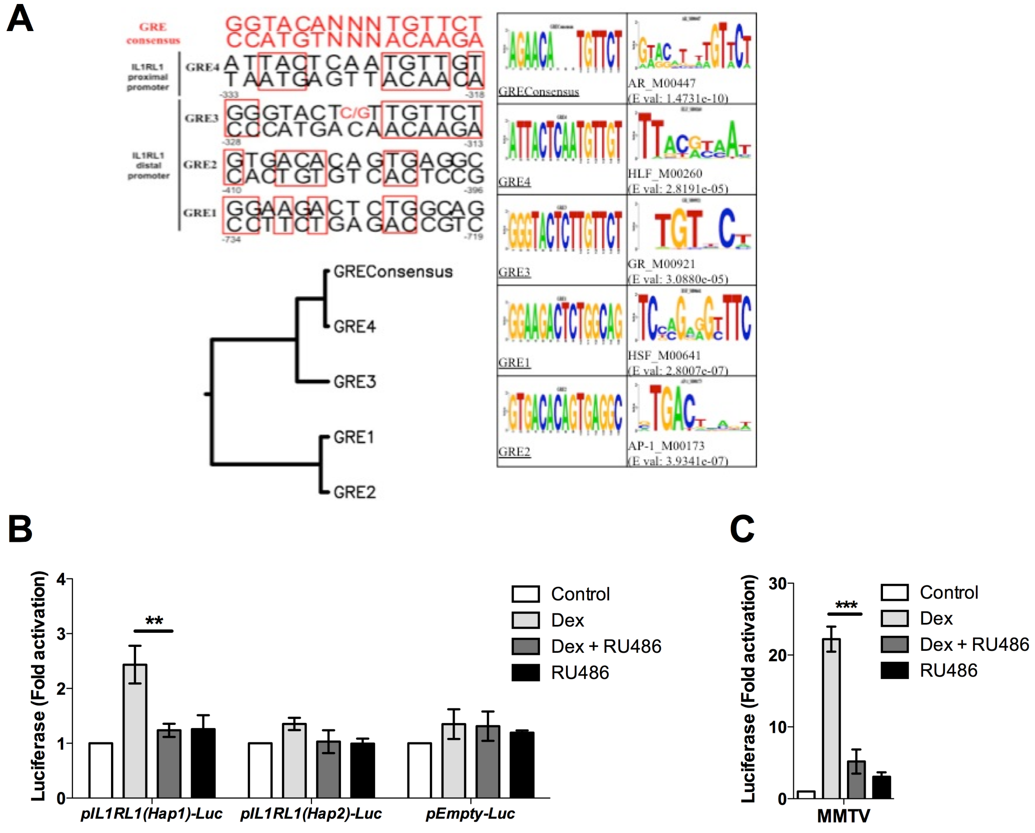


## Supplementary Figure 6

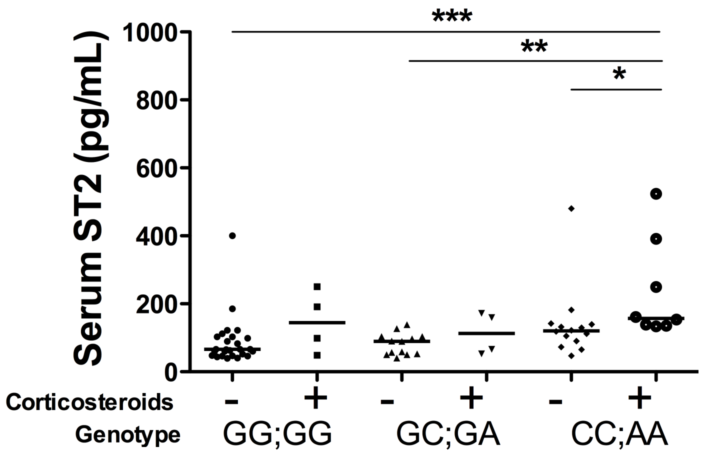

Supplement: Supplementary file 1 — Supplemental Information [file 41598_2017_10465_MOESM1_ESM.pdf]
